# Exostosis

**Published:** 1873-07

**Authors:** 


### EXOSTOSIS.

The subject of exostosis has difficulties which most practitioners have encountered. I do not mean to hint that it is difficult to diagnose a case of exostosis: that is a very simple matter. The questionable tooth has only to be drawn and examined? But if a diagnosis must be effected while the tooth yet retains its position in the alveolus, the aspect of the case is entirely changed. What was easy before may now prove one of the most perplexing and obstinate problems that the practitioner will encounter. We know that the patient suffers, and we are able to get descriptions of his pains, which though graphic are yet confused, if replete with detail, are yet contradictory. I have desired to call attention to this affection, and particularly to special points, connected with it.

In hastily writing the following questions, I have aimed to arouse thought and investigation among young practitioners. I have often thrown the interrogation into illogical forms, and purposely given a mistaken bias. I hope practitioners will communicate to the REGISTER their experience in treating this disease.

If we define exostosis to be an extension of ossific tissue by the addition of new matter upon its surface or within its substance, is our definition likely to be confounded with normal growth or with other forms of morbid growth?

To what forms of pathological tumefaction are the hard tissues subject, that are liable to be mistaken for exostosis?

In dental exostosis is the production of the new structure preceded by the formation of soft cartilage, into which the phosphates are deposited ? Is the new tissue a deposit upon the surface of the old, or is it an expansion of all parts of the old as a seeming result of the development and proliferation of the elementary parts ? In hypertrophy of the cement is the nutritive matter necessary to the increase of the part passed from the pulp through the dentinal tubuli, or is it furnished by the periosteum ?

It has been said that a continued irritation of the periosteum accompanies hypertrophy of the cement. Is this irritation always a concomitant symptom, and does it precede or follow the earliest hyper-ossific deposit ?

Was the observation of Thomas Bell, that the added substance in dental exostosis, is "harder, yellowish, and slightly transparent, (translucent)" ever confirmed by subsequent investigators ? If so, does the dentine in exostosed teeth share in this increased density, translucency and color ? Are other teeth in the same mouth usually well calcified, or in an exostosed tooth occasionally found among other teeth whose tissues have been starved for want of mineral substances ?

Does the nodulated or warty variety of exostosis depend upon the same or similiar causes as the cap-shaped variety in which the hypertrophied cement, covering the whole root, thins towards the neck of the tooth ?

Does exostosis depend upon constitutional conditions in any such manner as to associate it with calcification of the pulp ? Are the two affections frequently found together ? If so are there any other concomitant circumstances which we may safely set down as connected with them both ; such as peculiar temperaments, special diathesis, or particuliar habits as regards food and exercise, or marked systemic tendencies ? Do age or sex exert a modifying influence on diagnosis as regard frequency of presentation or severity of cases ?

Why does exostosis occur most often in bicuspids and molars ? Do additions to the cement occur after the pulp is devitalized ?

Do we often find exostosed teeth sensitive to percussion, painful to thermal changes or affected with inflammation of the pulp ? When exostosis is complicated with exposure o1 the pulp, or with sensitive dentine, how may the sympto ms of these three separate affections be discriminated and traced, each to its own proper cause. In what manner may we distinguish the neuralgia which arises from the lesion of a nerve from that which is due to exostosis ? D. C. H.

